# The Phytoestrogen Genistein Produces Similar Effects as 17*β*-Estradiol on Anxiety-Like Behavior in Rats at 12 Weeks after Ovariectomy

**DOI:** 10.1155/2017/9073816

**Published:** 2017-10-31

**Authors:** Juan Francisco Rodríguez-Landa, Jonathan Cueto-Escobedo, Abraham Puga-Olguín, Eduardo Rivadeneyra-Domínguez, Blandina Bernal-Morales, Emma Virginia Herrera-Huerta, Andrea Santos-Torres

**Affiliations:** ^1^Laboratorio de Neurofarmacología, Instituto de Neuroetología, Universidad Veracruzana, Xalapa, VER, Mexico; ^2^Facultad de Química Farmacéutica Biológica, Universidad Veracruzana, Xalapa, VER, Mexico; ^3^Posgrado en Neuroetología, Instituto de Neuroetología, Universidad Veracruzana, Xalapa, VER, Mexico; ^4^Facultad de Ciencias Químicas, Universidad Veracruzana, Orizaba, VER, Mexico

## Abstract

The phytoestrogen genistein produces anxiolytic-like effects in ovariectomized rats, which highlights its potential therapeutic effect in ameliorating anxiety in surgical menopausal women. However, no studies have directly compared the effects of identical doses of genistein and 17*β*-estradiol, the main estrogen used in hormone replacement therapy in menopausal women. The present study evaluated the anxiolytic-like effects of identical doses of genistein and 17*β*-estradiol (0.045, 0.09, and 0.18 mg/kg/7 days, s.c.) in a surgical menopause model in rats in the elevated plus maze and locomotor activity tests at 12 weeks after ovariectomy. Additionally, the participation of estrogen receptor-*β* in the anxiolytic-like effect of genistein and 17*β*-estradiol was explored by previous administration of the 5 mg/kg tamoxifen antagonist. Genistein and 17*β*-estradiol (0.09 and 0.18 mg/kg) similarly reduced anxiety-like behavior in the elevated plus maze and also increased the time spent grooming and rearing, without affecting crossing in locomotor activity test. These effects were blocked by tamoxifen. Present results indicate that the phytoestrogen genistein has a similar behavioral profile as 17*β*-estradiol in rats at 12 weeks after ovariectomy through action at the estrogen receptor-*β*. Thus genistein has potential for reducing anxiety-like behavior associated with low concentrations of ovarian hormones, which normally occurs during natural and surgical menopause.

## 1. Introduction

Natural or surgical menopause leads to low concentrations of estrogen and other hormones [1], among other physiological and psychological changes. The long-term reduction of estrogen predisposes women to the development of clinical signs, including skin atrophy [2], memory alterations [3], osteoporosis, cardiovascular disease [4], and anxiety and depression episodes [5, 6]. Hormone (e.g., estrogen) replacement therapy has been used for the management of typical physiological symptoms of natural and surgical menopause, and some beneficial effects on emotion and affect have been reported [7, 8]. Nonetheless, the long-term use of hormone replacement therapy can produce significant side effects, such as an increase in cerebrovascular accidents, deep vein thrombosis, venous thromboembolism [9, 10], and hormone-dependent breast cancer [11], thus limiting its use in some patients. Additionally, diets that are rich in soy-derived phytoestrogen are recommended as a therapeutic alternative to prevent some physiological symptoms that are associated with menopause [12–14] and reduce emotional symptoms, such as anxiety [15]. However, clinical research on the safety and efficacy of nondietary phytoestrogen therapy is scarce, which limits its use as a potential complementary therapeutic alternative for the treatment of typical menopause symptoms.

Preclinically, diets that have a high content of phytoestrogen (600 *µ*g/g) reduced anxiety-like behavior in animals with intact gonads compared with similar animals that were fed phytoestrogen-free diets [16, 17]. The phytoestrogen genistein that is contained in soja* (Glycine max)* and red clover (*Trifolium pratense* L.) produced beneficial effects on cardiovascular and osseous systems and exerted antitumor, anti-inflammatory [18], and anxiolytic-like effects in rats with the long-term absence of ovarian hormones produced by ovariectomy [19]. These effects appear to be related to the activation of estrogen receptor-*β* (ER*β*). Genistein has a structural conformation that is similar to 17*β*-estradiol, which allows it to be recognized by ER*β* [1, 20–22]. Therefore, genistein has been proposed as a possibly safe substitute for estradiol to ameliorate both physiological symptoms and anxiety associated with low concentrations of ovarian hormones, such as during natural and surgical menopause.

Ovariectomy in rats is used as a model of surgical menopause (equivalent to bilateral oophorectomy in women). Twelve weeks after ovariectomy, rats exhibited an increase in anxiety-like behavior compared with 3 weeks after ovariectomy [23]. In these rats, treatment with diazepam [23], 17*β*-estradiol [24, 25], and genistein significantly reduced anxiety-like behavior [19]. The anxiolytic-like effect of genistein was blocked by 5 mg/kg tamoxifen [26], which is an ER*β* antagonist at doses of 1–10 mg/kg [27, 28].

Although evidence indicates that 17*β*-estradiol and genistein reduce anxiety-like behavior in ovariectomized rats, the doses of genistein that are typically used are within the range of 0.25–1.0 mg/kg [19]. In contrast, the reported anxiolytic dose of 17*β*-estradiol in ovariectomized rats is 0.09 mg/kg [29, 30]. Therefore, it is unknown whether the same doses of 17*β*-estradiol and genistein produce similar effects on anxiety-like behavior in rats with the long-term absence of ovarian hormones produced by ovariectomy. The present study investigated whether phytoestrogen genistein has a behavioral profile that is equivalent to 17*β*-estradiol and, additionally, explored the participation of ER*β* in the behavioral effects produced by genistein and 17*β*-estradiol, which could contribute to design of alternative therapies for the treatment of anxiety symptoms associated with menopause.

## 2. Material and Methods

### 2.1. Ethics

All the experimental procedures were performed according to international ethical guidelines based on the National Institutes of Health Guide for the Care and Use of Laboratory Animals [31] and official Mexican guidelines (Especificaciones Técnicas para la Producción, Cuidado y Uso de Animales de Laboratorio) [32].

### 2.2. Animals

Adult female Wistar rats, weighing 200–250 g at the beginning of the experiments, were used. The rats were housed in Plexiglas cages (five rats per cage) under a 12/12 h light/dark cycle (light on at 7:00 AM) at an average temperature of 25°C ± 1°C with* ad libitum* access to water and food (Nutri-cubos Purina®, elaborated by Agribrands Purina México, Ciudad de México, México). Food guarantee analysis is as follows: 12.0% humidity, 3.0% fat, 7.0% ash, 1.0% calcium, 23.0% protein, 6.0% fiber, 47.4.0% NFE, and 0.6% phosphorus. Content is as follows: ground cereals, fish flour, cereal by-products, alfalfa, and cane molasses. All of the rats received their respective treatments 12 weeks after ovariectomy.

### 2.3. Ovariectomy

At 3 months of age, the rats were ovariectomized. Surgery was performed through an abdominal ventral incision under deep anesthesia with sodium pentobarbital (60 mg/kg, i.p., Cheminova de México, México City, México; Reg. SAGARPA Q-7048-044) and atropine sulfate (0.05 mg/kg, i.p., Sigma-Aldrich, St. Louis, MO, USA). The oviducts and ovaries were ligated and subsequently removed. The tissue area was carefully cleaned with benzalkonium chloride (Medipharm®, San Luis Río Colorado, Sonora, México), and then the muscle and skin were sutured separately. Analgesic-antipyretic medication (50 mg/kg Dipirona50®, i.m., Virbac Animal Health, Guadalajara, México) was administered to minimize postsurgical pain for 4 days after surgery. After surgery, the rats were returned to the housing facilities for 12 weeks to ensure the long-term absence of ovarian hormones and confirm an increase in anxiety-like behavior [19, 23]. Afterward, the rats were randomly assigned to the experimental groups, received their respective treatments, and underwent behavioral testing.

### 2.4. Experimental Groups and Dosage

#### 2.4.1. Effects of 17*β*-Estradiol and Genistein on Anxiety-Like Behavior

Fifty-six rats at 12 weeks after ovariectomy were assigned to seven independent groups (*n* = 8/group). The control group received the vehicle (Corn oil, Mazola® ACH Foods México, México City, México) in which genistein and estradiol were dissolved. The other six groups received 0.045, 0.09, and 0.18 mg/kg genistein (Sigma-Aldrich, St. Louis, MO, USA) or 17*β*-estradiol (Sigma-Aldrich, St. Louis, MO, USA). These doses were chosen because 0.09 mg/kg 17*β*-estradiol was shown to produce anxiolytic-like effects [29, 30]. To generate a dose-response curve, we included two additional doses: 0.5x and 2x the effective dose of estradiol that produces anxiolytic-like effects. Identical doses of both substances were used to compare their effects on anxiety-like behavior. All of the treatments were administered subcutaneously (s.c.) in a volume of 1 ml/kg for seven consecutive days. Sixty minutes after the last injection, the rats were evaluated in the elevated plus maze and then in the locomotor activity test.

#### 2.4.2. Antagonism of ER*β* on Anxiolytic-Like Effect of 17*β*-Estradiol and Genistein

Another set of forty-two rats at 12 weeks after ovariectomy were used to explore the participation of ER*β* in the effects produced by the minimum anxiolytic dose of genistein and 17*β*-estradiol (0.09 mg/kg) in the behavioral tests. This experiment included six independent groups (*n* = 7/group): vehicle (Vehicle); tamoxifen + vehicle group (T), vehicle + 17*β*-estradiol (17*β*-E2), 17*β*-estradiol + tamoxifen (17*β*-E2-T), vehicle + genistein (Genistein), and genistein + tamoxifen (Genistein-T) groups. The vehicle was corn oil in which tamoxifen, genistein, and estradiol were dissolved. Tamoxifen (Sigma-Aldrich, St. Louis, MO, USA) was administered at a dose of 5 mg/kg, which has been reported to readily penetrate blood-brain barrier and effectively block the anxiolytic-like effects of 17*β*-estradiol [27] and genistein [26] by antagonizing ER*β*. All of the treatments were administered subcutaneously (s.c.) in a volume of 1 mL/kg for seven consecutive days. Daily, tamoxifen or its vehicle was injected sixty minutes before injections of 17*β*-estradiol or genistein. Sixty minutes after the last injection at seventh day, the rats were evaluated in the elevated plus maze and then in the locomotor activity test.

### 2.5. Behavioral Tests

To evaluate the effects of the treatments, the rats were tested in the elevated plus maze (5 min) and then, approximately two minutes later, in a locomotor activity apparatus (5 min) as previously described [33, 34].

#### 2.5.1. Elevated Plus Maze

The apparatus consisted of two opposite open and closed arms set in a plus configuration. The apparatus was elevated 50 cm above the floor and illuminated at 40 lux. The dimensions of the open arms were 50 cm length × 10 cm width. The dimensions of the closed arms were 50 cm length × 10 cm width with 40 cm high walls. A digital video camera (Sony, DCR-SR42, 40x optical zoom, Carl Zeiss lens) was installed above the apparatus to record activity on a computer. Two independent observers measured the behavioral variables using* ex profeso* software until at least 95% agreement was reached for all of the measurements.

In the test session, the rats were placed in the center of the maze, facing an open arm. The following variables were evaluated: (i) time spent on the open arms, (ii) number of entries into the open arms, (iii) total number of arm entries (open arms + closed arms), and (iv) percentage of open arm entries ([open arm entries]/[total arm entries] × 100). These variables were selected based on previous studies, providing a reliable measure of experimental anxiety [35–37]. After the elevated plus maze test, the rats were evaluated in the locomotor activity test.

#### 2.5.2. Locomotor Activity Test

To evaluate the effects of the substances on spontaneous locomotor activity, grooming, and rearing, the rats were individually subjected to a 5-min locomotor activity test. An opaque Plexiglas cage (44 cm × 33 cm) with 20 cm high walls was used. The floor was delineated into 12 squares (11 cm × 11 cm). A digital video camera (Sony, DCR-SR42, 40x optical zoom, Carl Zeiss lens) was installed above the cage to record spontaneous activity. Two independent observers measured the behavioral variables using* ex profeso* software until at least 95% agreement was reached.

General locomotor activity was evaluated to discard or identify hypoactivity, hyperactivity, or no changes that were caused by the treatments that could interfere with behavior in the elevated plus maze.

At the beginning of the test, the rats were gently placed in one corner of the cage. The following variables were evaluated: (i) number of squares crossed (i.e., crossings; a crossing was recorded when the rat passed from one square to another with its rear legs), (ii) time (in seconds) spent rearing (rearing was recorded when the rat assumed a vertical posture relative to the cage floor), and (iii) time (in seconds) spent grooming, included paw licking, nose/face grooming (strokes along the snout), head washing (semicircular movements over the top of the head and behind the ears), body grooming/scratching (body fur licking and scratching the body with the hind paws), leg licking, and tail/genital grooming (licking of the genital area and tail) [38, 39].

After each test session, the elevated plus maze and locomotor activity apparatus were carefully cleaned with a 10% ethanol solution to remove the scent of the previous animals to avoid any possible influence on the spontaneous behavior of the subsequent rat [40].

### 2.6. Statistical Analysis

The data were analyzed using one-way analysis of variance (ANOVA), with treatment as the independent factor. Values of *p* ≤ 0.05 in the ANOVA were followed by the Student-Newman-Keuls* post hoc* test. The results are expressed as mean ± standard error.

## 3. Results

### 3.1. Effects of 17*β*-Estradiol and Genistein on Anxiety-Like Behavior

#### 3.1.1. Elevated Plus Maze

**Figure 1:**
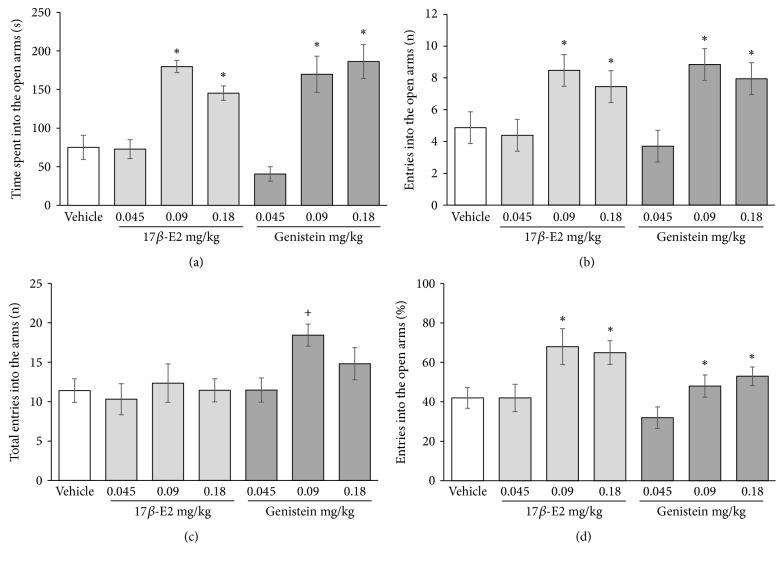
Effects of 17*β*-estradiol and genistein on anxiety-like behavior in the elevated plus maze. (a) Time spent on the open arms. (b) Number of entries into the open arms. (c) Total arm entries (open + closed). (d) Percentage of entries into the open arms. ^*∗*^*p* < 0.05 versus vehicle group. ^+^*p* < 0.05 versus 0.045 mg/kg 17*β*-estradiol, one-way ANOVA followed by Student-Newman-Keuls* post hoc* test.

The analysis revealed a significant effect of treatment on the time spent on the open arms (*F*_6,49_ = 15.802, *p* < 0.001). The* post hoc* test showed that rats that received 0.09 and 0.18 mg/kg 17*β*-estradiol and genistein spent a longer time on the open arms (*p* < 0.05) compared with the vehicle group and the lowest dose (0.045 mg/kg) of 17*β*-estradiol and genistein (Figure 1(a)). The analysis also revealed a significant effect of treatment on the number of entries into the open arms (*F*_6,49_ = 8.226, *p* < 0.001). The* post hoc* test indicated that 0.09 and 0.18 mg/kg 17*β*-estradiol and genistein increased the number of entries into the open arms compared with vehicle and 0.045 mg/kg 17*β*-estradiol and genistein (Figure 1(b)). The treatment factor also significantly affected the total number of entries into the arms (*F*_6,49_ = 8.236, *p* < 0.001). The* post hoc* test revealed that 0.09 mg/kg genistein increased the total number of arm entries compared with 0.045 mg/kg 17*β*-estradiol, but not with the vehicle group (Figure 1(c)). The treatment factor also significantly affected the percentage of entries into the open arms (*F*_6,49_ = 10.332, *p* < 0.001). The* post hoc* test revealed a significantly higher (*p* < 0.05) percentage of entries into the open arms in rats that were treated with 0.09 and 0.18 mg/kg 17*β*-estradiol and genistein compared with vehicle and 0.045 mg/kg 17*β*-estradiol and genistein (Figure 1(d)).

#### 3.1.2. Locomotor Activity

Table 1 shows the effect of treatments in the locomotor activity test. The statistical analysis revealed no effects of treatment on the number of crossings (*F*_6,49_ = 0.686, *p* = 0.662). The analysis of the time spent grooming revealed a significant effect of treatment (*F*_6,49_ = 10.332,* p* < 0.001). Rats that were treated with 0.09 and 0.18 mg/kg 17*β*-estradiol and genistein spent a longer time grooming (*p* < 0.05) compared with vehicle and 0.045 mg/kg 17*β*-estradiol or genistein. The analysis of the time spent rearing revealed a significant effect of treatment (*F*_6,49_ = 10.332, *p* < 0.001). The* post hoc* test showed that rats that were treated with 0.09 and 0.18 mg/kg 17*β*-estradiol and genistein spent a longer time rearing (*p* < 0.05) compared with 0.045 mg/kg genistein but not compared with the other treatment groups or vehicle.

### 3.2. Antagonism of ER*β* on Anxiolytic-Like Effect of 17*β*-Estradiol and Genistein

#### 3.2.1. Elevated Plus Maze

**Figure 2:**
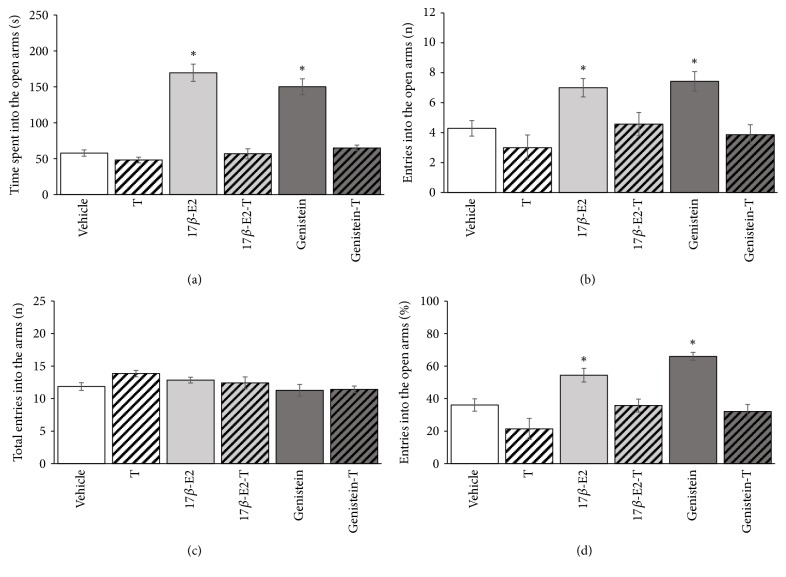
Involvement of the ER*β* on the anxiolytic-like effect of 17*β*-estradiol or genistein in elevated plus maze. (a) Time spent on the open arms. (b) Number of entries into the open arms. (c) Total arm entries (open + closed). (d) Percentage of entries into the open arms. ^*∗*^*p* < 0.05 versus Vehicle, T, 17*β*-E2-T, and Genistein-T. One-way ANOVA followed by Student-Newman-Keuls* post hoc* test. T, 5-mg/kg tamoxifen; 17*β*-E2, 0.09-mg/kg 17*β*-estradiol; Genistein 0.09 mg/kg.

The analysis revealed a significant effect of treatment on the time spent on the open arms (*F*_5,36_ = 44.049, *p* < 0.001). The* post hoc* test showed that rats that received 17*β*-estradiol and genistein spent a longer time on the open arms (*p* < 0.05) compared with the vehicle group, while rats that received 17*β*-estradiol plus tamoxifen or genistein plus tamoxifen had similar time on the open arms to vehicle, indicating that the effects of treatments were antagonized by tamoxifen (Figure 2(a)). The analysis also revealed a significant effect of treatment on the number of entries into the open arms (*F*_5,36_ = 6.692, *p* < 0.001). The* post hoc* test indicated that 17*β*-estradiol and genistein increased the number of entries into the open arms compared with vehicle group, and this effect was antagonized by tamoxifen (Figure 2(b)). The treatment did not significantly (*F*_5,36_ = 2.032, *p* < 0.097) affect the total number of entries (Figure 2(c)). The treatment significantly affected the percentage of entries into the open arms (*F*_5,36_ = 13.964, *p* < 0.001). The* post hoc* test revealed a higher (*p* < 0.05) percentage of entries into the open arms in rats that were treated with 17*β*-estradiol or genistein compared with vehicle, which was antagonized by previous administration of tamoxifen (Figure 2(d)). Tamoxifen* per se* is devoid of intrinsic activity on evaluated variables in the elevated plus maze.

#### 3.2.2. Locomotor Activity

Table 2 shows the effect of treatments in the locomotor activity test. The statistical analysis revealed no effects of treatment on the number of crossings (*F*_5,36_ = 2.176, *p* = 0.079). The analysis of the time spent in grooming revealed a significant effect of treatment (*F*_5,36_ = 17.951, *p* < 0.001). Rats that were treated with 17*β*-estradiol and genistein spent a longer time grooming (*p* < 0.05) compared with vehicle, but this effect was antagonized by tamoxifen. The analysis of the time spent in rearing revealed a significant effect of treatment (*F*_5,36_ = 9.010, *p* < 0.001). The* post hoc* test showed that rats that were treated with 17*β*-estradiol and genistein spent a longer time rearing (*p* < 0.05) compared with vehicle group, an effect antagonized by tamoxifen. Tamoxifen* per se* is devoid of intrinsic activity on evaluated variables in the locomotor activity test.

## 4. Discussion

The present study compared the effects of the same doses of the phytoestrogen genistein and 17*β*-estradiol on anxiety-like behavior in rats at 12 weeks after ovariectomy and then the participation of ER*β* in these effects. Doses of 0.09 and 0.18 mg/kg 17*β*-estradiol and genistein reduced anxiety-like behavior in the elevated plus maze (i.e., increased the time spent on the open arms, number of entries into the open arms, and percentage of entries into the open arms). 17*β*-Estradiol and genistein treatment did not affect the total number of arm entries in the elevated plus maze or the number of crossings in the locomotor activity test. The doses of 17*β*-estradiol and genistein that were anxiolytic in the elevated plus maze also increased the time spent grooming and rearing in the locomotor activity test. Previous administration of tamoxifen antagonized the anxiolytic-like effects produced by the minimal effective doses of 17*β*-estradiol and genistein (0.09 mg/kg) in the behavioral tests. These results indicate that genistein has a similar behavioral profile as 17*β*-estradiol in rats with the long-term absence of ovarian hormones, with participation of the ER*β*, suggesting that genistein may be an alternative therapy to ameliorate symptoms of anxiety that are associated with natural or surgical menopause.

During the biological development of women, several changes in physiological and emotional states occur when the levels of ovarian hormones or neurosteroids decrease during the premenstrual, postpartum, and menopause periods [41, 42]. Women with natural or surgical menopause are vulnerable to vasomotor symptoms, insomnia, depression, and anxiety that are caused by the downregulation of ovarian function that reduces the concentrations of steroid hormone, particularly estrogen [43–47]. Hormone replacement therapy is used to ameliorate such changes in menopausal women, but estrogen therapy is associated with possibly serious side effects, including breast cancer [48]. As an alternative, phytoestrogen is recommended to treat physiological and emotional symptoms in menopausal women, based on its anxiolytic effects in humans [49–53] and laboratory animals [19, 26, 54].

In preclinical studies of anxiety during menopause, rats are subjected to the long-term absence of ovarian hormones by ovariectomy. This procedure in rats resembles the physiological changes that are observed after bilateral oophorectomy in women and increases anxiety-like behavior [23, 55]. Treatment with estradiol [56, 57] or genistein [19] produces anxiolytic-like effects that are similar to diazepam. In the present study, we used the elevated plus maze to evaluate the effects of identical doses of 17*β*-estradiol and genistein on anxiety-like behavior in rats at 12 weeks after ovariectomy. The elevated plus maze is a useful model for studying anxiogenic- and anxiolytic-like effects in adult rats [35]. This model exploits the innate aversion of rats to open spaces and heights, which are stressful to the rat. Anxiolytic drugs usually increase the number of entries into and time spent on the open arms [35, 58]. In the present study, 17*β*-estradiol and genistein (0.09 and 0.18 mg/kg) produced similar behavioral effects as those mentioned above, thus demonstrating an anxiolytic-like effect.

General locomotor activity was also evaluated in the present study to discard or identify possible motor effects that could interfere with exploration of the elevated plus maze (i.e., hypoactivity or hyperactivity). The absence of changes in the number of crossings, together with the total entries to both arms, allowed us to discard the locomotor influence on behavior in the elevated plus maze. Therefore, the increases in the time spent on and entries into the open arms can be attributed to the emotional status of the animals, reflecting an anxiolytic-like effect of the treatments [35]. Rearing and grooming were also measured because these behaviors are indicators of the emotional state of rats that are exposed to novel environments [59]. In the present study, vehicle-treated ovariectomized rats exhibited the lowest level of grooming. These observations are similar to those in ovariectomized rats that presented high anxiety-like behavior in a light/dark model, which was restored by anxiolytic doses of diazepam [19]. In the present study, 17*β*-estradiol and genistein increased the time spent grooming, thus corroborating the anxiolytic-like effect that was observed in the elevated plus maze. The reduction of grooming behavior that is produced by exposure to severe stressors or the chronic absence of ovarian hormones is prevented by diazepam and others substances with well-characterized anxiolytic activity [19, 60, 61]. Additionally, rearing is considered a measure of exploration that is also influenced by “anxious” states in rats. Such animals spend less time exploring (reflected by rearing) and more time remaining quiet and alert to their surroundings [59]. Therefore, substances that have anxiolytic activity are presumed to increase rearing behavior, and such effects of 17*β*-estradiol and genistein were observed in the present study.

We found that identical doses of 17*β*-estradiol and genistein produced similar effects on anxiety-like behavior in ovariectomized rats. Treatment with 17*β*-estradiol at an anxiolytic dose of 0.09 mg/kg once weekly for 14 weeks increased the incidence and number of tumors in ovariectomized rats through ER*α* activation, whereas its anxiolytic-like effect is related to actions at ER*β* [29, 57]. Interestingly, genistein can bind both ER isoforms, although it binds ER*β* with 20-fold higher affinity compared with ER*α* [20]. Long-term phytoestrogen treatment may have beneficial estrogenic effects by acting on ER*β*, with minimal side effects because of minimal interactions with ER*α* compared with 17*β*-estradiol [62]. These findings suggest that genistein treatment may be an alternative to 17*β*-estradiol with a lower risk of producing side effects with long-term treatment in menopausal women [63].

In support, the present study explored the participation of ER*β* in the anxiolytic-like effect of 17*β*-estradiol and genistein by previous administration of 5 mg/kg tamoxifen. This dose of tamoxifen prevented the anxiolytic-like effects of both substances, indicating the participation of the ER*β* in the effects observed in 12-week ovariectomized rats, as previously reported [26, 29, 57]. Tamoxifen is not a pure estrogen receptor antagonist. It may act as agonist of ER*α* or antagonizing the ER*β* [64], which is dependent on the doses; this same effect occurs with raloxifene, another ER*β* antagonist [65]. In the present study, no significant effects of the tamoxifen doses* per se* were detected in elevated plus maze or locomotor activity test, discarding nonspecific behavioral effects on ER*α* or ER*β*. Altogether, results of antagonism in the present study suggest that tamoxifen used doses acted as an ER*β* antagonist, as reported previously in behavioral studies carried out by other authors [26–28].

On the other hand, it could be thought that rat food used in the present investigation might have some influence on the behavioral effects produced by 17*β*-estradiol and genistein, considering that soy and alfalfa, among other cereals, are the basis of protein in rodent chow and they contain phytoestrogen that may impact physiological processes in the organism [66, 67] and reduces anxiety-like behavior [16]. However, the effects of dietary isoflavones on anxiety-like behavior have been identified with isoflavones-rich diets compared with isoflavones-free diets, but not with standard diets [17]. The fact that in the present experiments all rats were fed with the same diet suggest that the effects produced by treatments are robust enough in long-term ovariectomized rats.

Although an anxiolytic-like effect was recently reported with the use of a standard diet in ovariectomized rats, the age of rats and the time of ovariectomy were not considered [68], and it is not comparable with rats ovariectomized at three months of age and then tested after 12 weeks after ovariectomy, at six months of age, used in the present study. Despite the fact that, in our first experiment, it was not possible to discard any physiological or behavioral effect produced by possible phytoestrogen content in the rat food, the second experiment discards any influence of food on anxiety-like behavior, because nonsignificant differences were found between vehicle-treated rats and rats treated with only tamoxifen (the ER*β* antagonist) fed with the same food. It is evident that the anxiolytic-like effect only was detected in rats treated with 17*β*-estradiol or genistein, but not in the vehicle-treated rats that also received the same food. This finding could discard any effect on anxiety-like behavior associated with dietary phytoestrogen, because if it had happened any difference in anxiety-like behavior would have been detected between vehicle and tamoxifen groups. Therefore, we consider that under our experimental conditions the possible content of phytoestrogen in food is not sufficient to interfere in the anxiolytic-like effect of 17*β*-estradiol or genistein in the ovariectomized rats. However, specific experiments could completely discard the effect of standard diets, isoflavones-free diets, and isoflavones-rich diets on anxiety-like behavior in rats with long-term absence of ovarian hormones produced 12 weeks after ovariectomy.

Finally, clinical evidence shows that moderate doses of phytoestrogen (~25 mg isoflavones) do not increase the risk of effects on cell proliferation [69]. A dose of 16 mg/kg did not produce adverse effects in menopausal women [54]. Additionally, doses of isoflavones, including genistein, which are lower than 100 mg/kg have been shown to be safe in breast cancer patients [70]. The effects and mechanism of action of phytoestrogen are dose-dependent [71], suggesting that genistein has a broader margin of safety at anxiolytic doses. However, controlled clinical trials are required to investigate genistein's possible pharmacological interactions, toxicity, and side effects to take advantage of its potential therapeutic anxiolytic effects.

In conclusion, the present study found that the phytoestrogen genistein exerted anxiolytic-like effects that were similar to 17*β*-estradiol by acting on ER*β* in rats with the long-term absence of ovarian hormones, that is, a surgical menopause model in rats. Genistein possibly has fewer side effects than 17*β*-estradiol when considering the low affinity of genistein for ER*α*.

## Figures and Tables

**Table 1:** Effects of 17*β*-estradiol, and genistein on crossing, grooming, and rearing in the locomotor activity test.

Groups	Crossing (n)	Grooming (s)	Rearing (s)
*Vehicle*	39.7 ± 3.7	12.3 ± 1.1	22.6 ± 2.6
*17β-Estradiol* (mg/kg)			
0.045	37.7 ± 2.6	11.8 ± 1.1	22.7 ± 2.9
0.09	40.5 ± 3.1	19.4 ± 2.7^*∗*^	29.7 ± 2.5^*∗*^
0.18	42.0 ± 1.8	23.0 ± 1.4^*∗*^	31.9 ± 2.4^*∗*^
*Genistein* (mg/kg)			
0.045	44.8 ± 1.8	10.5 ± 1.6	19.9 ± 3.2
0.09	39.6 ± 2.1	29.5 ± 1.4^*∗*^^+^	31.9 ± 2.0^*∗*^
0.18	39.5 ± 2.6	31.6 ± 2.4^*∗*^^+^	32.6 ± 2.2^*∗*^

^*∗*^ *p* < 0.05, versus vehicle and 0.045 mg/kg 17*β*-estradiol and genistein; ^+^*p* < 0.05, versus the same doses of 17*β*-estradiol. One-way ANOVA followed by Student-Newman-Keuls *post hoc* test.

**Table 2:** Effects of tamoxifen, 17*β*-estradiol, and genistein on crossing, grooming, and rearing in the locomotor activity test.

Groups	Crossing (n)	Grooming (s)	Rearing (s)
Vehicle	44.1 ± 1.7	10.3 ± 0.9	23.7 ± 2.0
T	52.5 ± 4.2	9.2 ± 1.1	21.5 ± 2.0
17*β*-E2	41.5 ± 2.4	22.6 ± 2.0^*∗*^	30.8 ± 2.3^*∗*^
17*β*-E2-T	47.2 ± 3.0	7.1 ± 0.8	18.3 ± 0.9
Genistein	41.7 ± 2.4	18.4 ± 2.2^*∗*^	33.0 ± 3.0^*∗*^
Genistein-T	50.1 ± 3.8	8.8 ± 0.8	17.7 ± 1.8

^*∗*^ *p* < 0.05, versus Vehicle, T, 17*β*-E2-T, and Genistein-T. One-way ANOVA followed by Student-Newman-Keuls *post hoc* test. T, 5 mg/kg tamoxifen; 17*β*-E2, 0.09 mg/kg 17*β*-estradiol; Genistein 0.09 mg/kg.
